# Graphene Oxide-Based Dispersive-Solid Phase Extraction for Preconcentration and Determination of Ampicillin Sodium and Clindamycin Hydrochloride Antibiotics in Environmental Water Samples Followed by HPLC-UV Detection

**DOI:** 10.22037/ijpr.2019.1100676

**Published:** 2019

**Authors:** Leila Mohammad nejad, Yaser Pashaei, Bahram Daraei, Mehdi Forouzesh, Maryam Shekarchi

**Affiliations:** a *Department of Toxicology, Faculty of Medical Sciences, Tarbiat Modares University Tehran, Iran. *; b *Young Researchers and Elite Club, Tehran Medical Sciences, Islamic Azad University, Tehran, Iran.*; c *Department of Toxicology and Pharmacology, Faculty of Pharmacy, Shahid Beheshti University of Medical Sciences, Tehran, Iran.*; d *Legal Medicine Research Center, Legal Medicine Organization, Tehran, Iran. *; e *Food and Drug Laboratory Research Center, Food and Drug Organization, MOH & ME, Tehran, Iran.*

**Keywords:** Graphene oxide nanosheets, Dispersive-solid phase extraction, Ampicillin sodium, Clindamycin hydrochloride, Adsorption isotherm, Environmental water samples, HPLC-UV

## Abstract

In this work, a reusable graphene oxide (GO) based dispersive-solid phase extraction (d-SPE) was synthesized and used for the analysis of trace ampicillin sodium (AMP) and clindamycin hydrochloride (CLI) in water samples followed by high performance liquid chromatography-UV detection (HPLC-UV). Batch experiments were conducted to investigate the effects of pH and volume of the sample solution, contact time, adsorption isotherms, temperature, and desorption conditions. The maximum adsorption capacities of AMP and CLI on GO nanosheets were found to be 33.33 mg g^-1^ and 47 mg g^-1^, respectively. The adsorption isotherm data can be well fitted by Temkin (AMP and CLI) and Freundlich (AMP), and the adsorption process followed the pseudo-second-order model. The thermodynamic parameters were calculated, indicated that the adsorption process of both analytes were spontaneous and exothermic. In addition, the d-SPE following HPLC analyses showed good linearity in the range of 0.5-200 ng mL^-1^ (R^2^= 0.999) for AMP and 1-200 ng mL^-1^ (R^2^= 0.999) for CLI, with LOD of 0.04 and 0.24 ng mL^-1^ for AMP and CLI, respectively. The percent of extraction recoveries, intra and inter-day precisions (expressed as RSD %, n = 3) were in the range of 96.4-101.6%, 2.2-3.0, and 3.7-4.7 for AMP as well as 94.2-98.6%, 2.2-3.8, and 3.5-4.6 for CLI, respectively. The preconcentration factor of 20 was achieved for both analytes. From these results, it can be concluded that the validated method is a simple, cost-effective and repeatable method for analysis of AMP and CLI in water samples and provide a new platform for antibiotics decontamination.

## Introduction

The aquatic ecosystem has been polluted by antibiotics due to their continuous input during the past few years (1). Ampicillin sodium (AMP) is a bactericidal amino penicillin and is active against many Gram (+) and Gram (-) bacteria. Clindamycin hydrochloride (CLI) is a semisynthetic derivative of lincomycin and belongs to the lincosamides class (2). Similar to the most antibiotics, large percentages of penicillins and lincosamides are excreted metabolized and unmetabolized by humans, animals and effluents of drug manufacturers to the surface and ground water (3). They have a high aqueous solubility and a long environmental half-life (4). Moreover, the presence of AMP and CLI in waste water and surface water, even at low concentrations, can lead to the development of antibiotic resistant bacteria. AMP and CLI have been measured in environmental water, at concentrations of 0.13 mg L^-1^ and 0.05 mg L^-1^, respectively (4-6). Since there are several questions that need to be answered on the occurrence of beta-lactam and lincosamides antibiotics in aquatic environment. Therefore, developing suitable validated methods for analysis of trace amount of antibiotics have attracted scientist’s attention. Until now the most common analytical technique for analysis of AMP and CLI in environmental water samples has been liquid chromatography (LC) with mass (MS) (7-9), tandem mass (MS-MS) (10-17) and ultraviolet (UV) (18-19) detection techniques. Capillary zone electrophoresis (CZE) with UV (20) and MS-MS (21) detection techniques has also been proposed for the analysis of these compounds. The expensive equipment required for LC-MS spectrometry methods, are not available in most of laboratories. However, compared to these methods, HPLC-UV is still broadly used due to its lower cost, simplicity, greater robustness and economy. A preconcentration step prior to the detection of AMP and CLI is imperative due to the low concentrations of antibiotics (ng L^-1^) and high complexity of the environmental waters. Until now, the solid phase extraction (SPE) technique for preconcentration of AMP and CLI in water has been applied due to factors such as high enrichment factor, good reproducibility, and selectivity (22, 23). Although SPE is applied widely, it has some shortcomings such as time-consuming sample loading step (especially for large sample volumes), solvent loss, large secondary wastes, and a need for complex and expensive equipment. The d-SPE is nowadays routinely utilized as clean up technique for the target enrichment due to signiﬁcantly reduced sample preparation time in comparison with conventional SPE. It can handle little samples, and consumes small quantity of solvent. Due to factors such as convenience, price, time reduction, and simplicity many researchers have applied this method of extraction using carbon nano materials (carbon nanotubes, multi-walled carbon nanotubes, activated carbon, and graphene oxide (GO)) as sorbent with high adsorption capacity for organic compounds (24-26). The novel properties of carbon based sorbents for determination of trace amount of antibiotics as SPE adsorbents have attracted the attention of scientist. These materials have attracted the attention of scientists because of remarkable properties such as high surface area, easy functionalization, and chemical stability which encourage them for determination of trace amount of antibiotics as SPE adsorbents. Graphene (G) based materials have been recently investigated for water treatment application including removal of dyes (27-29) and tetracyclines (30). Compared with other graphitic forms, the G shows many excellent advantages such as high surface area to weight (2630 m^2^ g^-1^) and low production cost (30-34). In this work, GO nanosheets as sorbents of beta-lactams and lincosamides were synthesized and characterized by energy dispersive X-ray spectroscopy (XRD), Fourier transform infrared spectroscopy (FT-IR), scanning electron microscopy (SEM). The effect of several variables on the determination of AMP and CLI such as adsorption kinetics (contact time and concentration), pH and volume of the sample solution, temperature, sorption and desorption rate, type and volume of eluent were investigated and optimized. Using this compound as d-SPE sorbent permits the sensitive, uncomplicated and inexpensive separation and determination of the AMP and CLI in aquatic environment samples which can be adopted by laboratories for quality control of water samples.

## Experimental

*Materials*

Ampicillin sodium (AMP) and clindamycin hydrochloride (CLI) reference standards were purchased from Fluka (Buchs SG, Switzerland). Analytical grade nitric acid (HNO_3_), hydrochloric acid (HCl), potassium chlorate (KClO_3_), and all HPLC grade solvents used in the chromatography analyses were obtained from Merck Co (Darmstadt, Germany). Water used in the experiments was prepared by water purification system (Purelab UHQ Elga, UK). Graphite powder (purity > 99%, 325 mesh) was purchased from Sigma-Aldrich (Buchs SG, Switzerland). The stock standard solutions of AMP and CLI (10 and 100 μg mL^-1^) were prepared in water and were stored at 4 °C. Working standards of AMP (0.5-200 ng mL^-1^) and CLI (1-200 ng mL^-1^) with different concentrations were prepared daily by appropriate dilution of the stock solution with phosphate buffer pH 6 and 7.6 for AMP and CLI, respectively. Tap-water samples were collected from a water tap in our laboratory (Tehran, Iran).

*Instrumentation*

Powder X-ray diffraction (XRD) patterns were obtained on a PANalytical X’Pert Pro MPD diffractometer with Cu-Kα radiation source (λ = 1.5406Å) at accelerating voltage 40 kV, 40 mA. FT-IR spectra of nanosheets were obtained on a Bomem MB 155S FT-IR spectrophotometer (Québec, Canada) using KBr pellets in the range of 400-4000 cm^-1^. The morphology and dimension analysis of the GO nanosheets were observed by scanning electron microscope (SEM) using a VEGA3-SB TESCAN SEM instrument (Brno, Czech Republic). The pH of mobile phase and all solutions were adjusted using Metrohm digital 744 pH meter (Metrohm Ltd, Herisau, Switzerland) equipped with a combined glass-calomel electrode. The HPLC experiment was performed using a Water Alliance (Milford, USA) HPLC system. The chromatographic system was composed of a 2996 Water UV detector, a multi solvent gradient Water pump, and a vacuum degasser. The UV spectra were collected across the range of 190-700 nm, extracting 230 nm, and 195 nm for AMP and CLI, respectively. Empower software was utilized for instrument control and data management. The column was ACE 5, C_18_ (4.6 mm × 250 mm, 5µm, Advance Chromatography Technologies Ltd, Aberdeen, UK). The column was eluted with AMP mobile phase (ammonium acetate buffer pH 6: acetonitrile, 83:17 v/v) in isocratic mode and CLI mobile phase (phosphate buffer pH 3: acetonitrile, 70:30 v/v) at flow rate of 1.0 mL min^-1^. The injection volume for all samples and standards was 20 µL (35-37).

*Synthesis of GO*

In order to synthesize GO, Staudenmaier method was used by some modification (38). Graphite powder (1g) was added slowly to a mixture of concentrated nitric acid (9 mL, 68%) and concentrated hydrochloric acid (18 mL, 37%).The reaction mixture temperature was kept at 0 ºC using an ice bath. Then, potassium chlorate (11 g) was gently added to the mixture under vigorous stirring in 1 h, while keeping the temperature less than 5 °C by cooling. The ice bath was removed and reaction was stirred at ambient temperature for 7days. For purification of the black pasty mixture, it was washed with deionized water until pH 7.The obtained precipitate was dried at 60 °C in vacuum oven. Then graphite oxide (0.2 g) in ethanol: water (100 mL, 50:50 v/v) was sonicated for about 2 h to obtain GO, and then was centrifuged at 10000 rpm for 5 min at ambient temperature. Finally, the obtained GO nanosheets were dried under vacuum for 12 h at 60 °C.

*Adsorption and desorption steps*

The adsorptions of AMP and CLI in an aqua-solution were investigated by batch experiments. GO (3 mg) was added to a series of tubes containing different concentrations of AMP (100 mL, in phosphate buffer pH 6) and CLI (100 mL, in phosphate buffer pH 7.6), separately. The obtained suspensions were shacked for 30 min (AMP) and 35 min (CLI), and then centrifuged at 4000 rpm in 25 °C for 5 min. Finally, the supernatant was filtered through a cellulose acetate filter (0.2 µm pore size, Advantec MFS Inc, CA, USA) and analyzed by HPLC-UV at the wavelength of 230 nm (AMP) and 195 nm (CLI), using a calibration curve built with different concentrations of analytes. The adsorption capacity (Q_e_, mg g^-1^) was calculated with the following equation:

$Q_{e}=\frac{{(C}_{0}-C_{t})\times V}{m}$

Where C_0_ and C_e_ are the initial and equilibrium concentrations of the drug in the aqueous solution (mg L^-1^), respectively. V is the volume of drug solution (mL), and m is the mass (mg) of sorbent. In desorption step the supernatant was removed and the remaining GO was washed with water (10 mL, twice), centrifuged at 4000 rpm and dried in vacuum oven, then was immersed in 5 mL of several solvents such as acetonitrile, methanol, and phosphate buffers pH 2-9. Afterward, the samples were shacked for 30 min (AMP and CLI) and centrifuged at 4000 rpm in 25 °C for 5 min. The supernatant was filtered through a cellulose acetate membrane syringe filter and 20 μL of filtrate was injected into the HPLC-UV system for analysis in triplicate (n = 3) for each concentration. In order to evaluate the influence of time and temperature on the adsorption of AMP and CLI, GO (3 mg) was added to a 100 mL of drug solution (AMP, 1 µg mL^-1^, pH 6 or CLI, 2 µg mL^-1^, pH 7.6) in different times (1-40 min) and temperatures (20-60 °C).

**Table 1:** Isotherm parameters for AMP and CLI sorption on GO

**Analytes**	**Langmuir**			**Freundlich**			**Temkin**		
	**Q (mg g ** **-1** **) ** **m**	**k (L mg** **-1** **)** **l**	**R** **2**	**k (L mg** **-1** **)** **f**	**1/n**	**R** **2**	**A**	**B**	**R** **2**
AMP	22.07	0.92	0.64	1.10	0.042	0.95	12359.26	3.65	0.97
CLI	0.64	6.29	0.34	1.10	0.11	0.04	7.55	24.0	0.98

**Table 2:** Thermodynamic parameters of AMP and CLI sorption on GO

	**∆G° (kJ mol** **-1** **)**		**∆S° (J mol** **-1 ** **K** **-1** **)**		**∆H° ( kJ mol** **-1** **)**	
**Temperature (K)**						
	**AMP**	**CLIN**	**AMP**	**CLIN**	**AMP**	**CLIN**
293	-29132896	-13728758				
298	-29329738	-13821519				
303	-29821844	-14053421				
313	-30806055	-14517226	9842.13	46380.48	-240.98	-135.65
323	-31790267	-14981031				
333	-32774478	-15444836				

**Table 3:** Kinetic parameters for AMP and CLI sorption on GO

**Analytes**	**Q** **e,exp**	**First-order model**			**Second-order model**		
		**K** **1**	**R** **2**	**Q** **e,cal**	**K** **2**	**R** **2**	**Q** **e,cal**
AMP	33.33	0.001	0.937	25.17	0.006	0.994	37.45
CLI	47.00	0.002	0.913	22.83	0.004	0.998	52.63

**Table 4:** Statistical and calibration parameters of AMP and CLI

Analytes	Equation	**LOD** *	**LOQ** *	**R** **2**	**Accuracy**		**Precision (RSD %)**		
					**Spiked value** *	**Found** *	**Recovery%±SD**	**Inter-day**	**Intra-day**
					5	5.08	101.6±3.7	4.7	3.0
AMP	y=135070x+38356	0.04	0.12	0.999	10	9.84	98.4±2.2	3.7	2.2
					20	19.28	96.4±6.2	4.6	2.7
					5	4.93	98.6±3.4	4.6	3.8
CLI	y=288762x-7485.5	0.24	0.71	0.999	10	9.78	97.8±1.3	3.5	2.2
					20	18.84	94.2±2.6	4.7	3.2
*ng mL-1									

**Table 5:** Comparison of the proposed method with other methods for the determination of AMP and CLI in water.

**Analytes**	**Method**	**Sample**	**Extraction method**	**Linear range**	**Recovery (%)**	**LOD**	**LOQ**	**RSD (%)**	**Ref.**
AMP	LC-MS	Sewage water	SPE-C2/ENV+	-	48	-	0.33 ng inj^-1^	-	7
		Sewage water	SPE-ENV+	-	50	-	60 h	-	8
		Tap water Ground water River water Sewage water	SPE-Carbograph 4	0-880 ng inj^-1^	77 83 81 99	3.3 ng inj^-1^	5 h 10 20 100	11 10 12 1	9
	LC -MS-MS^a^	Surface water Ground water	On line SPE-C18	5-500 h	24-61	0.5 h	-	9-24	15
		Waste water	SPE- Oasis HLB e	0.1-2 h	76-87	10-13^ h^	-	6.2-8.5	10
		Surface water	OASIS cartridge on top of the SDB cartridge	-	48(1 ng/L) 76(100 ng/L)	-	5 h	-	11
		Water	SPE-Strata-X	0.02-10 g	105	0.02 g	0.05 g	7.9	12
	CHPLC-DAD b	Well water River water	SPE- Alumina N	0.06–10^ g^	93-98 91-95	0.06^ g^	0.2 g	6.6-6.8	19
	HPLC-DAD	Swage water Ind. waste water	SPE-Oasis MAX SPE-Bond Elut C_18_	-	93 46	3.7 g 5.7	-	1	18
	CZE-DAD c	Well water River water Tap water	SPE- Oasis HLB	0.8-4^ g^	96	0.8^ g^	1.6 g	2.2	20
	CZE-MS-MS	Well water River water	SPE- Oasis HLB	0.24-20 g	92-98	0.24^ g^	0.69 g	3.3-3.5	21
	HPLC-UV	Tap water	d-SPE-GO	0.5-200^ g^	96.4-101.6	0.04^ g^	0.12 g	2.2-3.0	This work
CLI	LC-MS-MS LC-MS-MS	Waste water	SPE- Oasis HLB	0.05-10 g	83-86	-	0.05 g	8-10	13
		Surface water	OASIS cartridge on top of the SDB cartridge	-	103(1ng/L) 62(100ng/L)	-	1^ h^	-	11
		Surface water Ground Waters Waste water	SPE-Oasis HLB, tC18	0.0.27-2.5 g	87(0.25ng/L) 85(2.5ng/L)	0.027 h	0.1 g	11 4	14
	UHPLC-MS-MS d	Waste water	Chroma bonds HR-XX f	0.01-1000 g	91-102	0.005^ h^	0.01 g	-	16
		Hospital waste water Wastewater River water	SPE Oasis HLB		87-131	4.89 h 1.48 0.48	16.29^ h^ 4.93 1.61	5	17
	HPLC-UV	Tap water	d-SPE-GO	1-200 g	94.2-98.6	0.24 g	0.71 g	2.2-3.8	This work

, ^a^ Liquid chromatography- tandem mass

b Capillary high performance liquid chromatography-diod array detection,

c Capillary zone electrophoresis high performance liquid chromatography,

d Ultra high performance liquid chromatography-Mass-Mass,

e A copolymer made from a balanced ratio of hydrophilic N vinylpyrrolidone and lipophilic divinylbenzene

f , Polystyrene-divinylbenzene,

g ng mL^-1^,

h ng L^-1^

**Figure 1:**
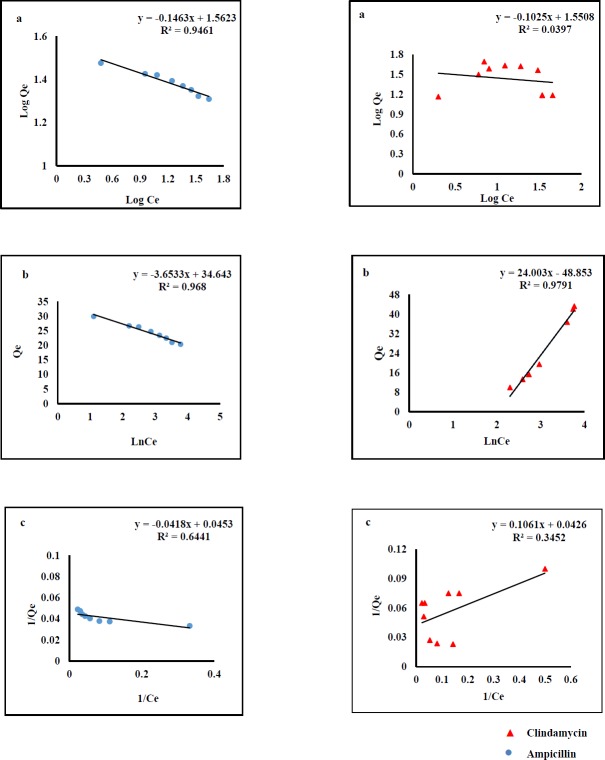
Adsorption kinetic models of AMP and CLI on GO, Freundlich (a) Temkin (b) and Langmuir (c)

**Figure 2:**
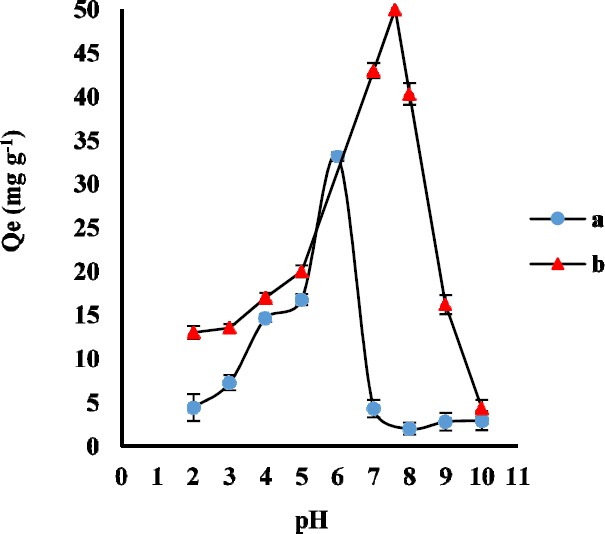
Effect of pH value (2-10) on adsorption capacity of AMP (a) and CLI (b) (n3 = for each point).Conditions: AMP concentration, 1 µg mL-1; CLI concentration, 2 µg mL-1; sample volume, 100 mL; extraction time, 30 min; GO amount, 3.0 mg

**Figure 3:**
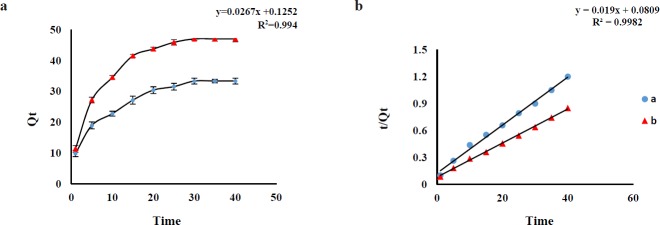
**a**. Effect of contact time on adsorption capacity of AMP (a) and CLI (b) on GO (n=3 for each point).Conditions: AMP concentration, 1 μg mL-1; CLI concentration, 2 μg mL-1; contact time 1-40 min; sample volume, 100 mL; GO amount, 3 mg; solvent, solution phosphate buffer pH 6 (AMP) and 7.6 (CLI). **b**, the pseudo-second-order kinetics model for adsorption of AMP (a) and CLI (b) on GO (n3 = for each point)

**Figure 4:**
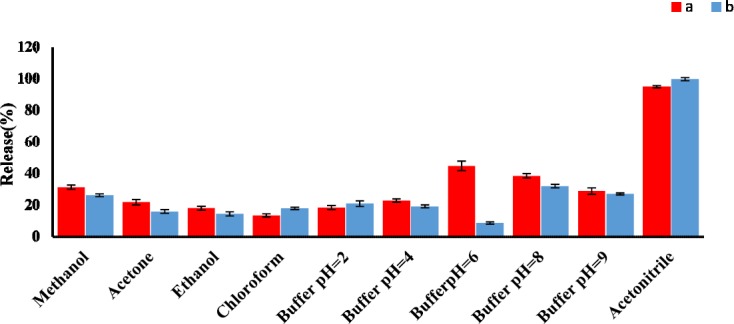
Effect of eluent type on the AMP (a) and CLI (b) release% (n3 = for each point). Conditions: AMP concentration, 1 µg mL-1; CLI concentration, 2 µg mL-1; sample volume, 100 mL; GO amount, 3 mg; Adsorption solvent, solution phosphate buffer pH 6 (AMP) and 7.6 (CLI); elution volume, 5 mL; desorption time, 30.0 min

**Figure 5:**
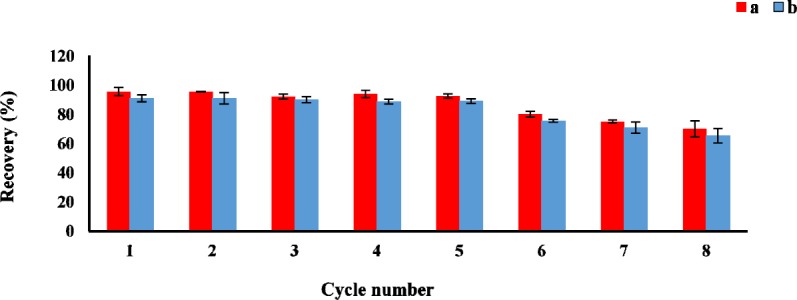
The reusability of the GO for AMP (a) and CLI (b) after eight uses (n3 = for each point). Conditions: AMP concentration, 1 µg mL-1; CLI concentration, 2 µg mL-1; sample volume, 100 mL; GO amount, 3 mg; Adsorption solvent, solution phosphate buffer pH 6 (AMP) and 7.6 (CLI); elution volume, 5 mL; desorption time, 30 min

**Figure 6:**
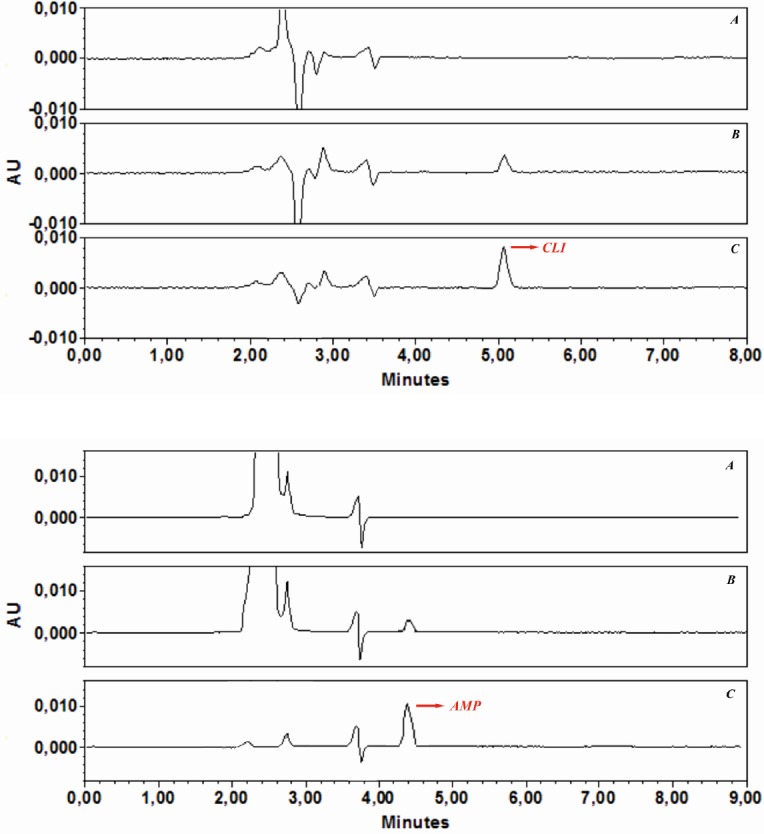
Typical chromatograms (HPLC) of (a) blank water sample before being spiked (b) after spiking with AMP (2 ng mL-1) and CLI (5 ng mL-1) and (c) preconcentration with GO under optimized conditions

*Method validation*

Method validation parameters, such as selectivity, linearity, precision, limit of detection (LOD), limit of quantification (LOQ), and recovery, were determined to confirm that the analytical methods are suitable for measurement of target analytes in water. The linearity of methods were indicated by analyzing a serial dilution of water (100 mL) spiked with standard to obtain different concentrations of AMP (0.5, 5, 10, 20, 50, 100, 150, and 200 ng mL^-1^) or CLI (1, 5, 10, 20, 50, 100, 150 and 200 ng mL^-1^). The samples were then submitted to the described procedure of preconcentration and chromatographic analysis. The calibration curves were constructed by plotting the average peak area versus concentration and the correlation coefficients were calculated. The ability of these methods to separate analytes in the presence of potential interferences was assessed by investigating the drug free water using the proposed analytical methods. Accuracy, Intra and inter-day precision (expressed as RSD%) of the proposed methods were evaluated at three concentration levels (5, 10 and 20 ng mL^-1^), and each concentration level was repeated three times both in the same day as well as in separate days. The extraction recoveries of drugs were performed by comparing the mean peak areas obtained from three replicate samples after preconcentration at three concentrations with the peak areas for pure compounds of the same concentrations in eluent solvent. The detection limits were determined as the concentration of drug giving a signal-to-noise ratio of 3 (S/N = 3). The quantification limits were defined as the lowest water concentrations of AMP and CLI quantified with a coefficient of variation of less than 20% (39).

## Results and Discussion

*Characterization of GO nanosheets*

The synthesized nanomaterial was characterized by FT-IR, XRD, and SEM. The XRD spectra of GO (Figure S1, supplementary information) reveals a high intensity peak at 2θ values of 12.81 with 6.9 Å interlayer corresponding to GO structure and very less intense peak at 2θ values of 26.48 Å interlayer corresponding to non-oxidation graphitic structure. The FT-IR spectrum of graphene oxide (Figure S2, supplementary information) confirms the successful oxidation of the graphite. The presence of different types of oxygen functionalities in graphene oxide were confirmed by a broad and wide peak at 3425 cm^-1^. The peak at 1721 cm^-1^ can be attributed to CO carboxylic. From Figure S3, it can be clearly seen that the GO nanosheets have a layered structure, with waves-like sheets, large surface area, and wrinkled edge.

*Adsorption isotherms of AMP and CLI on GO nanosheets*

Adsorption isotherms were used to describe AMP and CLI adsorption characteristics. The results clearly indicated that the adsorption capacity increases with rising the equilibrium concentration of the analytes. The equilibrium data were fitted by three isotherm models named Langmuir, Freundlich, and Temkin (Figure 1).The fitting parameters of AMP and CLI are scheduled in Table1. Langmuir model predicts the existence of a single layer of the adsorbate at the adsorbent surface. Freundlich model represents adsorption on heterogeneous surfaces. The chemical adsorption based on electrostatic interaction between adsorbent and adsorbate can be described by Temkin model (40). As it was observed in Figure 1, the regression values obtained from Temkin (AMP, R^2^=0.968; CLI, R^2^=0.979) and Freundlich models (AMP, R^2^=0.946) are better than those from Langmuir model. This fact reflects that there was an electrostatic interaction under the process of adsorption for both analytes. Cation- π bindings may happen between graphene π-electrons and amino group of AMP molecule (pKa values of 2.5 and 7.3) at the pH 6 and also positive early amine group in CLI structure (pKa values of 7.6 and 7.7) at the pH 7.6 (40-42). Considering here that aromatic ring in the molecular structure of AMP and CLI are present, we suggested π- π interactions may be also responsible for the adsorption of analytes onto GO nanosheets. The mathematical representations of the Langmuir Equ. (2), Freundlich Equ. (3), and Temkin Eq. (4) are given below:

$\frac{C_{e}}{Q_{e}}=\frac{C_{e}}{Q_{\max}}+\frac{1}{Q_{\max}\times k_{l}}$                     Equ, 1

Log Qe=Log Cen+log kf                    Equ, 2

$Q_{e}=\mathrm{Bln}C_{e}+\mathrm{B ln A}$                     Equ, 3

Where Q_max_ is the theoretical maximum adsorption capacity per unit weight of the adsorbent (mg g^-1^); K_l_, K_f_ and (A, B) are adsorption constants of Langmuir, Freundlich, and Temkin models, respectively, and n is the Freundlich linearity index.

*Effect of temperature*

The effect of temperature on the adsorption of AMP and CLI onto GO was investigated at 293, 298, 303, 313, 323, and 333 K. The adsorption energy changes of AMP and CLI on GO can be determined by thermodynamics analysis. The thermodynamics parameters such as standard enthalpy change (ΔH^0^, kJ mol^-1^), standard entropy change (ΔS^0^, J mol^-1^K^-1^), and standard Gibbs free energy change (ΔG^0^, kJ mol^-1^), were presented in Table 2. The standard Gibbs free energy changes are found to be negative at all temperatures, showing that the adsorption of AMP and CLI on GO was thermodynamically feasible process. At the same time, the values of standard Gibbs free energy changes were relatively close to each other with an increase from 293 to 333 K, indicating the effect of temperature on adsorption of AMP and CLI was slight. 

The standard enthalpy change value was calculated to be negative which means that the adsorption of AMP and CLI on GO was an exothermic process. In addition, the positive change of standard entropy reveals the randomness adsorption process (43). The dependence on temperature of sorption of AMP and CLI by GO are evaluated by the following equations:

$∆G=∆\mathrm{H{}^{\circ}}-\left(T\times ∆\mathrm{S{}^{\circ}}\right)$                     Equ, 4

$\mathrm{Ln}K_{C}=\frac{∆\mathrm{S{}^{\circ}}}{R}-\frac{∆\mathrm{H{}^{\circ}}}{\mathrm{RT}}$                     Equ, 5 

$K_{c}=\frac{Q_{e}}{C_{e}}$                     Equ, 6

Where ΔH, ΔS, ΔG, T, R, and K_C_ are the enthalpy, entropy, Gibbs free energy, absolute temperature (K), ideal gas constant (8.314 J mol ^-1^), and equilibrium constant, respectively. Plots of log K_C_ against 1/T give numerical values of ΔH and ΔS from the slope and intercept, respectively.

*Optimization of GO based d-SPE parameters*

In order to obtain the adsorption capacity (Q_e_) the main parameters, including sample pH, contact time, eluent solvent, elution volume, and desorption time were studied and optimized. Besides, the reusability of the sorbent was also discussed. All the tests were performed in triplicate at ambient temperature and the means of the results were used for optimization of the method.

*Effect of pH*

Generally, the pH of the sample solution determines the molecular or ionic form of the analytes and the surface charge of the sorbent. AMP structure has the primary amine and carboxyl groups with pK_a _7.3 and 2.7, respectively. Moreover, CLI structure has two proton binding sites with pK_a _7.6 and 7.7. In order to evaluate the effect of sample pH on the adsorption of AMP and CLI on GO, the appropriate volumes of phosphate buffer were used to adjust the pH values between 2 to 10. The experiment was carried out for adsorption of 2 ng mL^-1 ^AMP and 5 ng mL^-1 ^CLI on 3 mg GO as adsorbent for a contact time of 30 and 35 min, respectively. As it was shown in Figure 2, the Q_e_ significantly increased from pH 2 to 6 for AMP and pH 2 to 7.6 for CLI, and reached the maximum value at pH 6.0 and 7.6 due to strong attraction between the negative charge of GO functional groups (such as -OH and -COOH) and the protonated AMP and CLI. Therefore, buffer solution of pH 6 and 7.6 were selected as the working pH for the following AMP and CLI experimental studies, respectively.

*Effect of contact time*

In order to optimize the best contact time, the effect of shaking time on the Q_e_ was investigated in the range of 1 to 40 min (AMP and CLI) at ambient temperature. The results revealed an increasing trend of AMP and CLI adsorption over time and the adsorption equilibrium was achieved after about 30 min for AMP and 35 min for CLI with desired pH. The adsorption capacities (Q_e_, mg g ^-1^) of the AMP (at a time of 30 min) and CLI (at a time of 35 min) were about 32.70 and 49.63 mg g ^-1^, respectively, which are sufficient for the adsorption of trace amounts of AMP and CLI from sample (Figure 3a). Pseudo-first-order and pseudo-second-order adsorption kinetic models were used to analyze the experimental data for the AMP and CLI adsorption onto GO at different time. The fitting parameters are scheduled in Table 3. AMP and CLI adsorption processes on GO were fitted by pseudo-second-order kinetic model (Figure 3b), which also were confirmed according to the correlation coefficient values (AMP, R^2^=0.994; CLI, R^2^=0.998) for pseudo-second model, higher than that of pseudo-first-order (AMP, R^2^=0.922; CLI, R^2^=0.961), suggesting that the adsorption system may be controlled by chemical reaction and adsorption capacity is proportional to the number of active sites on adsorbent surface(30). The comparison between the experimental (exp) adsorption capacity (AMP, Q_e exp_: 33.10 mg g^-1^; CLI, Q_e exp_: 50.11 mg g^-1^) value and the calculated (cal) adsorption capacity (AMP, Q_e cal_: 37.1 mg g^-1^; CLI, Q_e cal_: 53.47 mg g^-1^) value shows that Q_e cal_ values are very close to Q_e exp_ values for the pseudo-second-order kinetics. The pseudo-first-order (Equ. 8) and -second-order (Equ.9) adsorption kinetic models are expressed as follows:

$\mathrm{Log}\left(Q_{e}-Q_{t}\right)=\mathrm{Log}Q_{e}-\frac{k_{1}}{2.303}\times t$                     Equ, 7

$\frac{t}{Q_{t}}=\frac{1}{k_{2\times }Q_{e}}+\frac{1}{Q_{e}\times t}$                    Equ, 8

Where Q_e_ and Q_t_ are the adsorption amount (mg g^-1^) at equilibrium and time t, respectively, K_1_ and K_2_ are equilibrium rate constant of pseudo-first and -second- order sorption.

*Effect of type and volume of eluent*

A good desorbing solvent in d-SPE should effectively elute the adsorbate with the lowest volume without damaging the nature of the adsorbent surface. In the present study, different solvents such as acetonitrile, methanol, and different phosphate buffer pH 2 to 9, were tested to obtain the best eluent for the removal of AMP and CLI and their release efficiencies were evaluated. The results shown in Figure 4 clearly indicated that acetonitrile had the highest release percent in comparison with other solvents. Under the same experimental conditions, the effect of eluent volume on release efficiency of AMP and CLI were investigated in the volume range of 1 to 5 mL. Our results indicated that an acceptable preconcentration factor and release can be obtained using acetonitrile (5 mL) as eluent for both analytes (Figure S4, supplementary information).

*Effect of desorption time*

Desorption time is another main factor influencing the recovery of the AMP and CLI. Hence, it was optimized by increasing the shake time from 1 to 60 min. The influence of desorption time on the release percent is shown in Figure S5. The experimental results indicated that 30 min was enough for eluting the adsorbed AMP and CLI from the surface of the GO sorbent, and it was selected as the appropriate desorption time for both drugs.

*Reusability of the sorbent*

The reusability is one of the important factor for evaluating the stability and efficiency of the adsorbent. The sorption-desorption batch cycles were repeated 8 times by the aforesaid procedure. After each cycle, the GO as sorbent was isolated and washed three times with acetonitrile, then air-dried at room temperature before reusing in the next cycle. As shown in Figure 5, the GO could be effectively reused at least 8 times without a significant loss of the sorption capacity.

*Effect of sample volume and validation parameters*

In the analysis of real samples, the sample volume is one of the important parameters influencing the pre concentration factor. Therefore, the effect of sample volume on quantitative adsorption of spiked AMP and CLI in tap-water was investigated, and all water samples were also filtered through a cellulose acetate membrane syringe filter. For this purpose, GO (3 mg) was dispersed in different sample volumes (20, 50, 100, 250, 500, 750 and 1000 mL) containing appropriate volumes phosphate buffer solution pH 6 for AMP and pH 7.6 for CLI Then, the obtained suspensions were shacked 30 min (AMP) and 35 min (CLIN), respectively. The samples were then submitted to the procedure of extraction and chromatographic analysis described above. The recoveries were found to be stable until 100 mL. After determination of preconcentration factor for both analytes, in order to demonstrate the applicability of the developed GO-based d-SPE-HPLC-UV method for analysis of AMP and CLI in the real samples, it was applied to the extraction and determination of both analytes in the water under the optimized conditions. The typical chromatograms of (a) a blank water sample before being spiked with AMP and CLI, (b) after spiking with AMP (2 ng mL^-1^) and CLI (5 ng mL^-1^), and (c) preconcentration with GO under optimized conditions are shown in Figure 6a, b, and c. The comparison between the purity threshold and angle in Empower software revealed that the methods were specific for AMP and CLI. Analytical performance under the optimum conditions described, the calibration curves were linear over a concentration range of AMP (0.5-200 ng mL^-1^) and CLI (1-200 ng mL^-1^). Statistical and calibration parameters are shown in Table 4. The LOQ were found to be 0.12 (AMP) and 0.71 (CLI) ng mL^-1^. As it was shown in Table 4, the low values of the relative standard deviation proved that the method was precise and repeatable. The preconcentration factor of 20 was obtained by the ratio of the slopes of the linear section of the calibration curve before and after preconcentration. These results demonstrated that the GO-based d-SPE combined with HPLC-UV analysis was reliable for measurement of trace amount of AMP and CLI in water samples.

*Comparison of GO-based d-SPE with other reported methods*

A comparison between the performance characteristics of the proposed method and previous reported methods for the analysis of AMP and CLI in aqueous samples are summarized in Table 5. The most commonly analytical methods applied to the determination of AMP and CLI in water samples are LC-MS and LC-MS-MS (7-17), but these methods require expensive equipment and expert staff which are not readily available in many laboratories. Additionally, it is necessary to dry the final eluent of SPE before separation into the chromatographic system in the majority of previously reported methods (7-17, 19, 20, 21). The separation process in this method was highly efficient without the need for evaporation step. The reported LC-MS and LC-MS-MS with evaporation technique required a longer time for equilibrium to be established. As shown in Table 5, the GO-based d-SPE-HPLC-UV methods have a little higher LOQ than some of the reported methods, which can be attributed to the sensitivity of the detection technique and evaporation process. These methods can be applicable in monitoring of AMP and CLI in environmental water samples. The developed methods in this work for AMP and CLI analysis in water samples showed a good recovery and repeatable results.

## Conclusion

In this paper, a GO-based d-SPE clean-up combined with HPLC-UV has been successfully developed and employed as a new approach for the separation and determination of trace amount of AMP and CLI in water samples. The adsorption of AMP and CLI to the GO nanosheets could be described by Temkin and Freundlich-type adsorption model. The pseudo-second-order kinetic model best described the adsorption behavior of AMP and CLI on GO. The method was appropriated to the trace AMP and CLI determination at three levels, and the recoveries for the spiked tap-water samples with AMP and CLI were 96.4-101.6% and 94.2-98.6% for concentrations of 5, 10, and 20 ng mL^-1^, respectively, and also the method was convenient, cost-effective, environmentally friendly, with a very low consumption of sorbent and eluent solvent. Furthermore, the results revealed that the developed method offers satisfactory repeatability, low LOQ, high preconcentration factor, and acceptable recoveries. In summary, this method has great potential for the monitoring AMP and CLI in water samples.
