# Supplementary material for: Graphene Oxide-Based Dispersive-Solid Phase Extraction for Preconcentration and Determination of Ampicillin Sodium and Clindamycin Hydrochloride Antibiotics in Environmental Water Samples Followed by HPLC-UV Detection
Source: Iran J Pharm Res. 2019 Spring;18(2):642–57. doi: 10.22037/ijpr.2019.1100676 (PMC6706713; doi:10.22037/ijpr.2019.1100676)
Supplement: Supplement [file ijpr-18-642-s001.pdf]

Supplementary Materials for

Graphene Oxide-Based Dispersive-Solid Phase Extraction for Preconcentration and Determination of Ampicillin Sodium and Clindamycin Hydrochloride Antibiotics in Environmental Water Samples Followed by HPLC-UV Detection

Leila Mohammad nejad, Yaser Pashaei, Bahram Daraei*, Mehdi Forouzesh and Maryam Shekarchi*

To whom correspondence should be addressed: shekarchim@yahoo.com; bdaraei@sbmu.ac.ir

Volume 18, issue 2 (Spring 2019)

This PDF file includes:

Figures S1 to S5

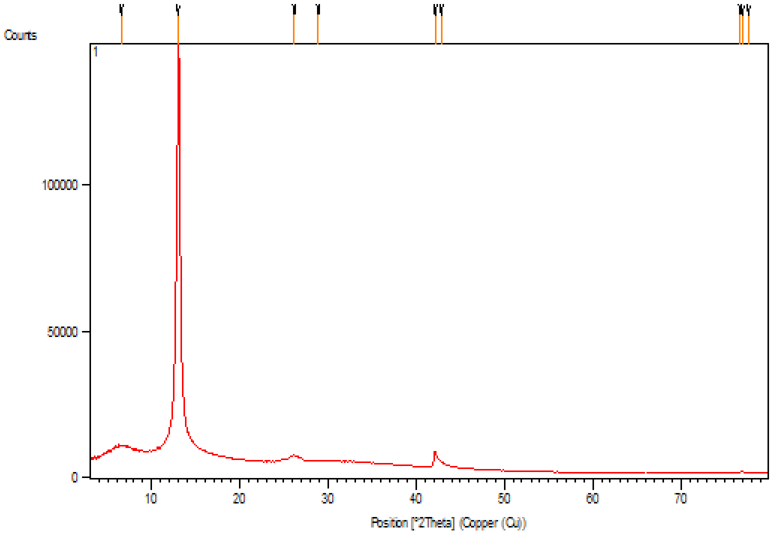

Figure S1. X-ray diffraction pattern of GO.

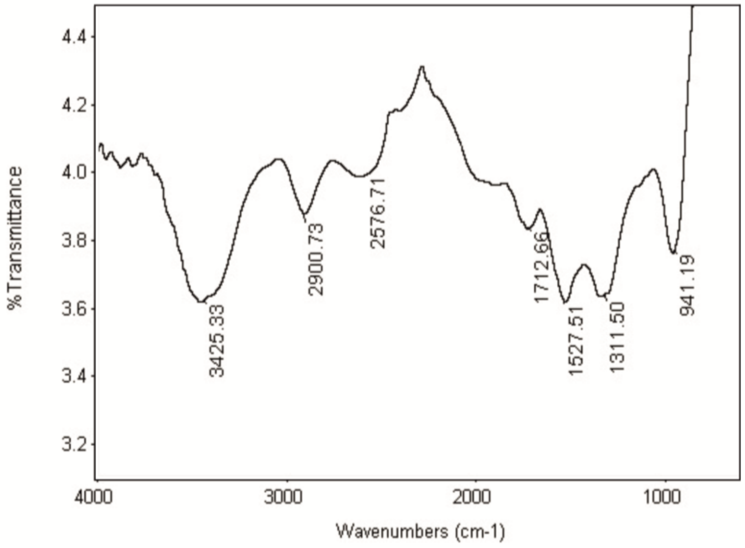

Figure S2. FT-IR spectra of GO.

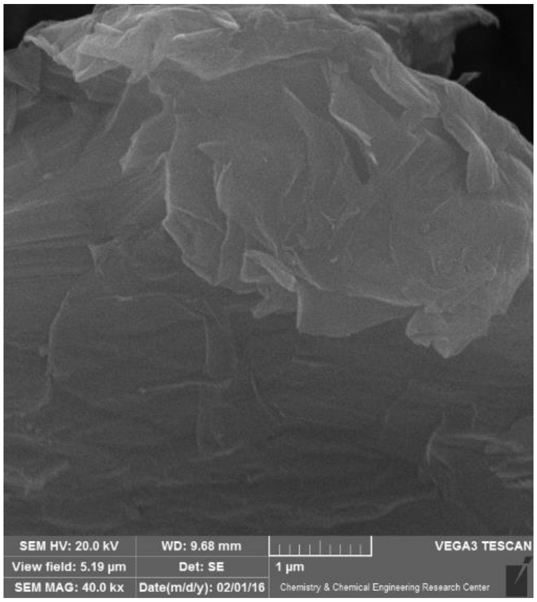

Figure S3. SEM image of GO.

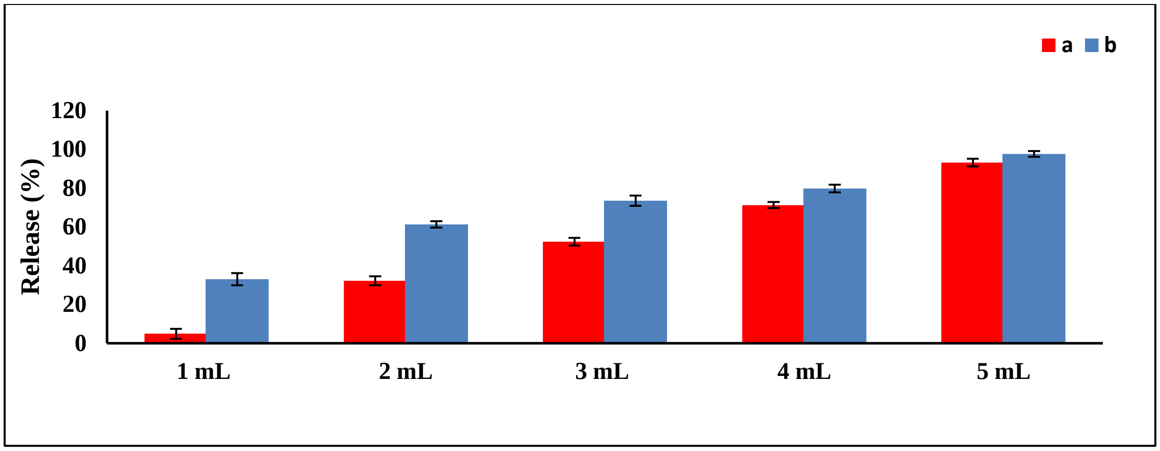

Figure S4. Effect of acetonitrile volume on the AMP (a) and CLI (b) Release% (n = 3 for each point). Conditions: AMP concentration, 1 µg mL^{-1}; CLI concentration, 2 µg mL^{-1}; GO amount, 3 mg; elution solvent, solution acetonitrile; elution volume, (1-5) mL; desorption time, 30 min.

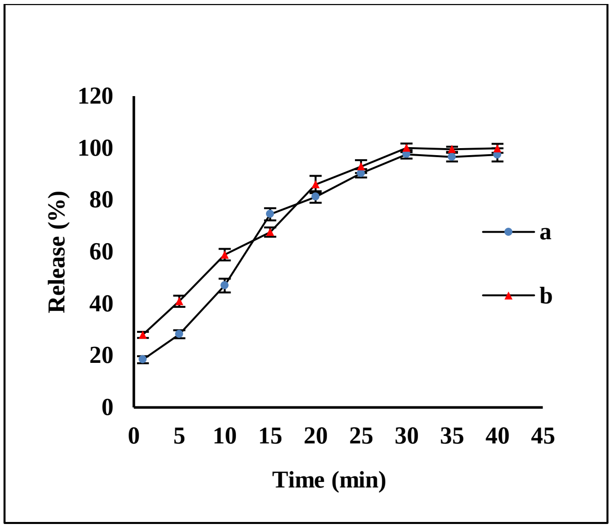

Figure S5. Effect of desorption time on the AMP (a) and CLI (b) Release% (n=3 for each point).Conditions: AMP concentration, 1 µg mL^{-1}; CLI concentration, 2 µg mL^{-1}; pH value, pH 6 (AMP) and 7.6 (CLI); extraction time, 30 min (AMP) and 35 min (CLI); sorbent amount, 3 mg; elution solvent, acetonitrile; elution volume, 5 mL; desorption time, 1-45 min.
